# A mixture model approach to multiple testing for the genetic analysis of gene expression

**DOI:** 10.1186/1753-6561-1-s1-s141

**Published:** 2007-12-18

**Authors:** Cyril Dalmasso, Joseph Pickrell, Marianne Tuefferd, Emmanuelle Génin, Catherine Bourgain, Philippe Broët

**Affiliations:** 1 JE 2492 Universite Paris-Sud, Hôpital Paul Brousse – Batiment 15/16, 16 Avenue Paul Vaillant Couturier, Villejuif CEDEX 94807, France; 2 INSERM UMR-S 535, Universite Paris-Sud, Villejuif F94807, France; 3 Department of Human Genetics, The University of Chicago, 920 East 58th Street, Chicago, Illinois 60637, USA

## Abstract

With the availability of very dense genome-wide maps of markers, multiple testing has become a major difficulty for genetic studies. In this context, the false-discovery rate (FDR) and related criteria are widely used. Here, we propose a finite mixture model to estimate the local FDR (lFDR), the FDR, and the false non-discovery rate (FNR) in variance-component linkage analysis. Our parametric approach allows empirical estimation of an appropriate null distribution. The contribution of our model to estimation of FDR and related criteria is illustrated on the microarray expression profiles data set provided by the Genetic Analysis Workshop 15 Problem 1.

## Background

In the context of genetic studies for which high-density genetic maps are now widely available, a major multiple testing problem arises due to the large number of statistical tests that are performed simultaneously. In a recent study, Morley et al. [1] analysed microarray gene-expression data together with genome-wide single nucleotide-polymorphism (SNP) genotyping in 14 three-generation families to localize the genetic determinants underlying gene-expression variability (data provided for Genetic Analysis Workshop 15 (GAW 15) Problem 1). For the genome-wide linkage analysis, the authors calculated a non-parametric Haseman-Elston statistic and used the genome-wide significance thresholds proposed by Lander and Kruglyak [2] to identify linked loci. Thus, they controlled the classical family-wise error rate (FWER), i.e., the probability of falsely rejecting at least one null hypothesis.

Although the FWER is the oldest extension of the classical type I error rate, FWER-based procedures are often too conservative, particularly when numerous hypotheses are being tested [3]. As an alternative and less stringent error criterion, Benjamini and Hochberg introduced, in their seminal paper published in 1995 [4], the false-discovery rate (FDR), which is defined as the expected proportion of false discoveries among all discoveries (here, a discovery refers to a rejected null hypothesis). The opposing criterion, the false non-discovery rate (FNR), corresponds to the expected proportion of false non-discoveries among all the non-rejected null hypotheses [5].

More recently, Efron et al. introduced the local FDR (lFDR) [6], which can be interpreted as a variant of the Benjamini and Hochberg's FDR that gives each tested null hypothesis its own "measure of significance". While the FDR is defined for a whole rejection region, the lFDR is defined as the probability that a null hypothesis is true conditional on a particular value of the test statistic. As discussed by Efron [7], the local nature of the lFDR is advantageous for interpreting results from individual test statistics. Moreover, the FDR can be estimated directly from the lFDR [6].

Efron proposed an empirical Bayes' procedure [7,8] to estimate the lFDR without any assumption about the distribution under the alternative hypothesis. From this procedure, only an upper bound estimate can be obtained for the lFDR and, indirectly, a lower bound for the FNR. One important feature of this approach is that it considers an empirical rather than theoretical null distribution. Indeed, as noted by Efron, these distributions may be very different and strong arguments support using the empirical one in genetic studies for which extensive data are available [5].

In this work, and for variance-component linkage analysis, we introduced a two-component mixture model based approach that allows estimation of lFDR, FDR, and FNR. We illustrate the contribution of our model to the analysis of real GAW15 data. Our results highlight the importance of correctly estimating the null distribution through the proposed mixture model based approach.

## Methods

Consider the variance-component linkage analysis between a particular phenotype (here, the expression level of a defined gene) and a specific marker. The null hypothesis of no linkage (additive genetic variance due to the studied quantitative trait locus (QTL) equals zero) is tested by comparing the likelihood of this restricted model with that of a model in which the variance is estimated. Under the null hypothesis, the theoretical asymptotic distribution of the likelihood-ratio statistic *X *is a 50:50 mixture of a *χ*^2 ^and a point mass at 0 [9]. When testing *n *markers, *n *likelihood-ratio statistics *X*_*i*_, (*i *= 1,...,*n*) are available, with each *X*_*i *_following either the null or the alternative distribution.

For modeling of the marginal distribution of *X*, we consider the following two-component mixture model, in which the marginal cumulative distribution *F*_*X *_of *X *is:

*F*_*X*_(*x*) = *ω*_1 _× {*θ *× 1_{*X*=0} _+ (1 - *θ*) × *F*_1_(*x*|*α*_1_, *β*_1_)} + *ω*_2 _× *F*_2_(*x*|*α*_2_, *β*_2_),

where *ω*_*c *_is the mixing proportions for the *c *components (*c *= 1, 2; *ω*_*c *_∈ [0, 1]; *ω*_1 _+ *ω*_2 _= 1). Here, *c *= 1 corresponds to the null hypothesis component and *c *= 2 to the alternative hypothesis component, respectively. The parameter *θ *∈ [0, 1] is the weight of the point mass at 0 for the null hypothesis component.

In this model, the conditional distributions *F*_*c*_(*x*|*α*_*c*_, *β*_*c*_) are gamma distributions with parameters *α*_*c *_and *β*_*c*_, where *α*_*c *_is the mean and *α*_*c*_/*β*_*c *_the variance of the distribution. Here, we impose that *α*_1 _<*α*_2_.

As discussed in the Background, the empirical distribution under the null hypothesis can be very different from the theoretical distribution [8]. Therefore, we decided to not consider theoretical values (*θ *= 1/2, *α*_1 _= 1, and *β*_1 _= 1/2) for the first component distribution parameters but rather to estimate them. For the second component, we used a gamma distribution, which represents a convenient and parsimonious way to model the non-null distribution.

Parameters of interest are inferred by sampling from their joint posterior distributions using Monte Carlo Markov chain (MCMC) samplers implemented in WinBUGS software [9]. All results presented correspond to 25,000 sweeps of MCMC algorithms following a burn-in period of 25,000 sweeps (period required to achieve algorithm stability). Convergence is checked by visual inspection of the curve of the plots for the different parameters of the mixtures.

For each marker, the posterior probabilities of belonging to the null hypothesis can be estimated directly from the algorithm output, using empirical averages. These probabilities are natural estimates of the lFDR for each marker. They can be used to compute model-based estimates of the observed FDR and FNR (conditionally to the data) [10,11].

## Results

We started from the cell intensity files (*.CEL) obtained from the GeneChip^® ^Human Genome Focus Array Hgfocus [12] that provide gene-expression measurements of 8794 probe sets for 276 samples. We normalized and summarized those measurements using the robust multi-array average (RMA) method proposed by Irizarry et al. [13]. A multipoint variance-component linkage analysis was performed with MERLIN [14] on the normalized phenotypes using all 194 individuals from the 14 Centre d'Etude du Polymorphisme Humain (CEPH) families and the 2819 autosomal SNP data. Using the proposed mixture model, we then estimated the lFDR at each marker, and FDR and FNR. Here, we present only the results obtained for the following 10 genes discussed in the article by Morley et al. [1]: *CHI3L2*, *DDX17*, *PSPHL*, *IL16*, *HOMER1*, *ALG6*, *CBR1*, *TNFRSF11A*, *TGIF*, and *DSCR2*.

**Figure 1:**
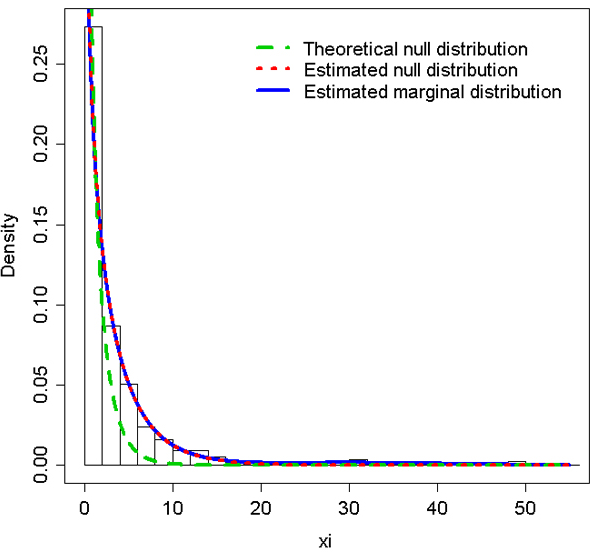
Histogram distribution of the (non-null) observed likelihood ratio statistic, theoretical null hypothesis density, and marginal and null hypothesis densities estimated from the mixture model for the *DDX17 *gene.

Table 1 gives the estimated parameters of the two-component mixture model for the expression of each of the 10 genes (phenotypes). The estimated values of the null distribution parameters differed markedly from the theoretical values. For the 10 selected genes, the maximal differences between the theoretical and empirical values were: 0.11 for *θ *(*PSPHL*), 1.96 for *α*_1 _(*DDX17*), and 1.08 for *β*_1 _(*ALG6*). For example, Figure 1 illustrates the histogram distribution of the (non-null) observed likelihood-ratio statistic *X*, and superimposed theoretical null hypothesis, marginal and null hypothesis densities estimated from the mixture model for the *DDX17 *gene. The marked difference between the theoretical and estimated null distributions strongly supports the use of the estimated null distribution rather than the theoretical one. As noted by Efron [8], these differences can substantially affect any simultaneous inference (including FDR estimation and FWER control). It is worth noting that when the FWER is controlled at 5% with a classical Bonferroni procedure, the *p*-values for the *DDX17 *gene calculated from the theoretical null distribution yielded 52 significant results, while the *p*-values calculated from the estimated null distribution gave only 13 significant results. In this example, considering the theoretical null distribution clearly tended to overestimate the number of significant results.

**Table 1:** Estimated parameters of the two-component mixture model for each of the ten genes analyzed

Gene	*θ*	*α*_1_	*β*_1_	*α*_2_	*β*_2_
CHI3L2	0.41	1.72	0.41	40.40	0.47
*DDX17*	0.47	2.96	0.24	33.90	0.32
*PSPHL*	0.39	2.76	0.25	63.04	0.73
*IL16*	0.50	1.08	0.90	5.14	1.85
*HOMER1*	0.45	1.02	0.92	3.36	1.25
*ALG6*	0.53	0.79	1.58	9.71	2.77
*CBR1*	0.41	0.74	1.37	2.42	2.15
*TNFRSF11A*	0.52	1.27	0.68	7.34	2.97
*TGIF*	0.54	0.70	1.35	3.96	1.47
*DSCR2*	0.52	0.83	1.17	3.57	1.38

**Figure 2:**
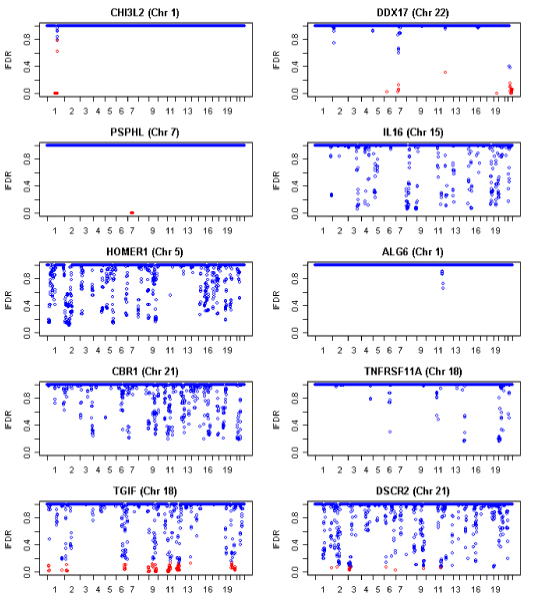
**Estimated posterior probabilities (lFDR) for the 10 selected genes along the 22 chromosomes**. Significant results at FDR threshold 0.05 are plotted in red.

Summary statistics calculated from the full output of the MCMC algorithm (after discarding the burn-in samples) provide information on the posterior probabilities of belonging to the null hypothesis component. Using these estimates, probabilistic classification of the data (in terms of discoveries and non-discoveries) can be obtained concomitantly with the estimations of FDR and FNR [10,11]. Herein, we decided to consider as discoveries (linkage) the markers with posterior probabilities below a threshold value, which can be different for each phenotype and was chosen to ensure 5% FDR. Figure 2 shows the estimated posterior probabilities (equivalent to the lFDR) along the 22 chromosomes for the 10 phenotypes. Meanwhile, the estimated FNR ranged from 23% (*PSPHL*) to 28% (*HOMER1*) (data not shown). The selected markers with an lFDR estimate below the defined threshold are plotted in red. These selected markers differed substantially from those obtained by Morley et al. [1]. For example, we found multiple *cis*-acting and *trans*-acting regulators for *DDX7 *and *IL16*, while Morley et al. [1] found only *cis*-acting regulators for these genes.

However, it is difficult to directly compare the two approaches because the selection strategies rely on completely different criteria. Moreover, it is worth noting that while the Bonferroni procedure depends on the order of the *p*-values, our procedure depends on the order of the posterior probability (lFDR) values, and the two can be completely different.

## Conclusion

Herein we described a mixture model based approach to estimate FDR, FNR, and lFDR in the context of variance component linkage analyses. This approach allows the selection process to take into account both false positives and false negatives. Moreover, it provides an estimate of the empirical null distribution, which is a key component for any simultaneous inference procedure.

Indeed, in many situations, the empirical null distribution deviates from the theoretical one [8], leading to incorrect statistical inferences and resulting decisions. Traditional estimating methods in linkage analysis used simulation approaches in which marker alleles were randomly dropped from the genealogies. When markers are numerous or pedigrees are complex, that method can become very burdensome, with computations requiring several days of running time. New genetic studies for which large amounts of data are available open new opportunities by allowing the estimation of appropriate null and alternative densities without resorting to simulations. Hence, our approach is much easier to handle because examination of each of the different phenotypes analysed required less than 1 hour of computer time. It is important to note that this approach can be extended by incorporating different null distribution parameters for a set of phenotypes in a single model. In conclusion, we think that new insights on linkage analysis using genome-wide technologies might emerge from a mixture model-based approach.

## Competing interests

The author(s) declare that they have no competing interests.
